# The impact of Oncotype DX testing on adjuvant chemotherapy decision making in 1–3 node positive breast cancer

**DOI:** 10.1002/cnr2.1546

**Published:** 2021-10-19

**Authors:** Yogeshkumar Malam, Mohamed Rabie, Konstantinos Geropantas, Susanna Alexander, Simon Pain, Mina Youssef

**Affiliations:** ^1^ Department of Breast Surgery Norfolk and Norwich University Hospital Trust Norwich UK; ^2^ Faculty of Medicine Ain Shams University Cairo Egypt; ^3^ Department of Oncology Norfolk and Norwich University Hospital Trust Norwich UK; ^4^ Surgical Oncology National Cancer Institute, Cairo University Cairo Egypt

**Keywords:** adjuvant chemotherapy, breast cancer, gene based assay, node positive, Oncotype DX

## Abstract

**Background:**

Oncotype DX testing has reduced the use of adjuvant chemotherapy in node‐negative early breast cancer but less is known about its impact in node positive patients.

**Aim:**

This study aimed to investigate the impact of Oncotype DX gene assay testing on the decision to offer adjuvant chemotherapy in oestrogen positive, human epidermal growth factor receptor 2 negative, 1–3 lymph node positive patients.

**Methods:**

Retrospective review of all node positive patients who underwent Oncotype DX testing at a single centre. Clinicopathological data, as well as estimated survival benefit data (from the PREDICT tool), was evaluated by a multidisciplinary group of surgeons and oncologists. Treatment decisions based on clinicopathological data were compared to recurrence scores (RS). A cut off RS > 30 was used to offer adjuvant chemotherapy.

**Results:**

The 69 patients were identified, of which 9 (13%) had an RS > 30 and assigned a high‐genomic risk of recurrence. The 32 patients (46.4%) were offered adjuvant chemotherapy. Overall based on the use of the RS, the decision to offer adjuvant chemotherapy changed in 36% of patients, and ultimately 24 patients (34.7%) would have been spared chemotherapy.

**Conclusion:**

Using clinicopathological data alone to make decisions regarding adjuvant chemotherapy in node positive breast cancer leads to overtreatment. Additional information on tumour biology as assessed by the Oncotype DX RS helps to select those patients who will benefit from adjuvant chemotherapy and spare patients from unnecessary chemotherapy.

## 1 INTRODUCTION

Recent advances in our understanding of early breast cancer have shown it to be a heterogeneous disease with tumour biology becoming a more important factor in determining clinical course, response to treatment and long‐term survival.^1^, ^2^, ^3^

Traditionally the decision for adjuvant chemotherapy in early breast cancer has largely relied on prognostic information based on clinicopathological features of the patient and tumour. More recently, gene expression profiling which captures tumour biology has been increasingly used to aid clinical decision‐making.

The Oncotype DX test (Genomic Health, Redwood City, CA, USA) is a reverse transcriptase polymerase chain reaction based assay which measures the expression of a panel of 21 genes (16 cancer‐related, five reference) and generates a recurrence score (RS)–a value from 0 to 100.^4^ This score reveals underlying tumour biology and also represents an individualised estimate of the risk of disease recurrence and prognosis.

Previous work with ER+, HER2‐, node‐negative patients has shown that the RS accurately predicts the benefit of adjuvant chemotherapy helping identify patients who would not benefit from the addition of chemotherapy to adjuvant endocrine therapy. In the landmark TAILORx trial, it was shown that ER+, HER2‐, node‐negative women with RS 0–15, and women above age 50 with RS 0–25 did not benefit from adjuvant chemotherapy with no difference in survival compared to endocrine therapy alone.^5^ Multiple studies have shown the use of Oncotype DX testing in the node‐negative setting has changed treatment decisions in up to 30% of patients.^6^, ^7^, ^8^

More recently, attention has turned to the utility of the RS in node positive patients, to try to identify patients who would benefit from adjuvant chemotherapy.^9^

Retrospective data from the SWOG‐8814 trial found that only ER+, HER2‐, node positive patients identified as high risk, which was defined as a RS >30, benefitted from the addition of adjuvant chemotherapy compared to tamoxifen alone.^10^ There was no benefit from the addition of adjuvant chemotherapy in low risk patients (RS < 18) or for the intermediate (RS 18–30) group, although this trial was underpowered to detect a subtle difference.

The prospective WSG/planB trial showed that node positive patients with a RS < 11 had excellent (94.3%) 5‐year disease free survival without chemotherapy.^11^ Patients with intermediate‐risk (defined as RS 12–25) underwent chemotherapy and showed a similar 5‐year disease free survival of 94.3% suggesting that there may be some benefit of chemotherapy in this group. The ongoing RxPONDER trial is evaluating the role of adjuvant chemotherapy in node positive patients with a RS ≤25.^9^ Patients will be randomised to hormonal therapy with or without adjuvant chemotherapy. The estimated completion date for the trial is 2022.^12^

From these studies, it is clear that gene expression profiling has a role in guiding treatment selection of node positive patients to identify those patients who would not benefit from chemotherapy.^9^, ^13^ More evidence to support this is still needed.

Decisions about breast cancer treatment are made by a multidisciplinary team (MDT) including breast surgeons and oncologists. In addition to clinicopathological data, standard UK practice is to use, a prognostication tool such as PREDICT to aid decision making.^14^ This model uses various clinicopathological features to derive estimates for 10‐year survival and quantify the benefits of the addition of adjuvant chemotherapy.^15^

Our breast cancer MDT is one of the early adopters of Oncotype DX testing in a selected group of node positive patients since 2014. Patients were selected for Oncotype DX testing if the MDT felt there was a benefit in further information to aid decision making of adjuvant chemotherapy.

The current study aims to examine the real‐life impact of Oncotype DX testing on the decision to offer adjuvant chemotherapy to ER+ HER2‐ node positive patients compared to decisions made by a simulated MDT based solely on clinicopathological data.

## 2 METHODS

A retrospective cohort study was conducted at Norfolk and Norwich University Hospitals NHS Trust, a tertiary teaching hospital treating approximately 700 new breast cancer patients per year. All patients with ER+, HER2‐, 1–3 lymph node positive breast cancer who underwent Oncotype DX testing between July 2014 and April 2020 were identified from a prospectively maintained database. All patients underwent appropriate surgery to remove the primary breast tumour and either sentinel lymph node biopsy or axillary clearance. Patients were selected to undergo Oncotype testing by a contemporaneous MDT if it was felt that additional information from the test would guide decisions to offer or omit adjuvant chemotherapy in patients with intermediate‐risk. Patients with micrometastases or isolated tumour cells were excluded. Patients with four or more lymph nodes and/or distant metastases were excluded.

Clinical data were collected for each patient including age, multifocality of disease, tumour histology, tumour grade, size of tumour, presence of lymphovascular invasion, ER positivity/Allred Quick score, number of positive nodes and details of treatment to date. Furthermore, estimates of 10‐year survival and the added benefit of adjuvant chemotherapy were recorded for all patients using the online PREDICT tool (breast.predict.nhs.uk).

RS calculated from the Oncotype DX test (Genomic Health, Redwood City, CA, USA) performed on each tumour were also collected.

A simulated MDT panel composed of three members (two oncologists, one surgeon) reviewed the available clinicopathological for each patient and estimated survival data, before making a recommendation of whether to offer adjuvant chemotherapy or not. PREDICT scores were available to the simulated MDT members. The members of the MDT panel were blinded to the RS for each patient.

The simulated MDT decision to offer adjuvant chemotherapy was compared to the RS recommendation for chemotherapy. A RS of greater than 30 was used to select high‐risk patients who would benefit from chemotherapy based on previous studies.^10^

The two decisions were compared for each patient aiming to examine the impact of the Oncotype DX test in real life.

### 2.1 Statistical analysis

Statistical analysis was performed using Microsoft Excel (Microsoft Corporation, Redmond, VA). A *p* value of <.05 was taken as statistical significance. The Mann–Whitney U test was used for continuous variables, whilst Fisher's exact test and Chi‐squared test was used for categorical variables.

### 2.2 Cost analysis

A cost analysis to assess the financial impact of Oncotype testing was performed using previously published sources. The current list price to the NHS for the Oncotype DX test is £2580.^16^ The full cost of a course of chemotherapy, including drug costs, delivery costs and toxicity, is estimated to be between £3866.17 and £4863.70 depending on regimen.^16^, ^17^ The cost of performing the Oncotype DX test on all patients in the cohort was compared to the potential savings of omitting chemotherapy in patients with a low RS. Further subgroup cost analysis excluding patients with favourable PREDICT scores (<2% predicted survival benefit) was also performed.

## 3 RESULTS

A total of 69 patients with ER+, HER2‐, and 1–3 lymph node positive breast cancer were referred for Oncotype DX testing during the period between July 2014 and April 2020. The median age was 61 years (range, 33–76 years). Fifty‐five patients (79.7%) had one positive lymph node, 12 patients (17.3%) had two positive lymph nodes and two patients had three positive lymph nodes. The median Oncotype DX RS was 16 with a range of 0–63.

**FIGURE 1:**
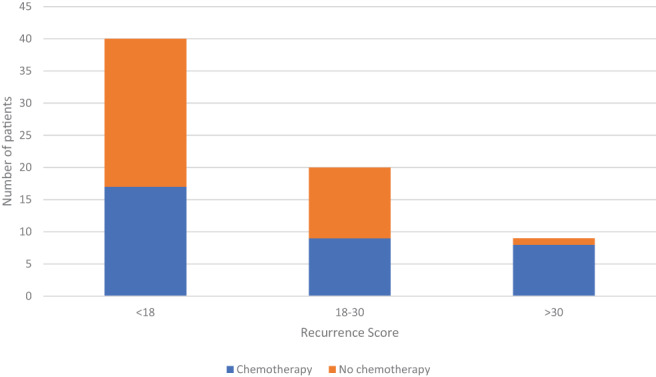
Graph showing the distribution of patients by RS into low, intermediate and high risk groups. Breakdown of each group by the number of patients recommended to undergo adjuvant chemotherapy based on the multidisciplinary team (MDT) decision

In 32 patients (46.4%), the simulated MDT recommended adjuvant chemotherapy. Figure 1 shows the number of patients offered adjuvant chemotherapy by the MDT based on stratification by genomic risk based on traditional RS cut‐offs. Using a threshold RS > 30, nine patients (13%) were considered as high genomic risk patients who would benefit from adjuvant chemotherapy, whereas patients with an RS < 30 were considered low risk based on current evidence.

Table 1 summarises clinicopathological data for included patients and their PREDICT scores.

**TABLE 1:** Table summarising the clinicopathological data high recurrence scores (RS) (>30) and low RS (<30) genomic risk groups

Characteristic	Whole cohort	Recurrence score 0–30	Recurrence score > 30	*p* value
*N*	69	60	9	
Median age (range)	61 (33–76)	59 (33–76)	63 (39–74)	.0819^a^
Detected by screening	31	30	1	.0353^b^
Detected by symptoms	38	30	8	
Grade				
Grade 1	19	19	0	.0003^c^
Grade 2	35	33	2	
Grade 3	15	8	7	
Median tumour size in mm (range)	19 (8–65)	18 (8–65)	25 (13–28)	0.3735^a^
Lymphovascular invasion present	26	20	6	.0718^b^
Lymphovascular invasion absent	43	40	3	
Median nodes sampled (range)	3(1–32)	3 (1–23)	3 (1–32)	0.3077^a^
Nodes positive (range)	1(1–3)	1 (1–3)	1 (1–2)	0.6031^a^
PREDICT score benefit				
<2%	14	14	0	.0195^c^
2%–5%	40	36	4	
>5%	15	10	5	

*Note*: A *p* value <.05 was taken as statistical significance.

^a^ Mann–Whitney U test.

^b^ Fisher's exact test.

^c^ Chi‐squared test.

Patients in the high genomic risk group (RS > 30) were more likely to present with symptoms (*p* = .0353) and with a higher tumour grade (*p* = .0003). No patients in the high‐genomic risk group had Grade 1 tumours. Estimated additional 10‐year overall survival benefit as calculated using the PREDICT tool was moderately correlated with RS (r^2^ = 0.44, *p* = .02). All patients with a PREDICT score indicating >5% survival benefit with chemotherapy were recommended to undergo adjuvant chemotherapy by the simulated MDT (Supplementary Figure S1).

Concordance between the MDT decision and RS recommendation regarding the decision to offer adjuvant chemotherapy to patients or not was reached in 44 patients (63.7%). Conversely, discordance between the MDT decision and RS was observed in 25 patients (36.3%). In the vast majority of these cases (24/25 patients) the simulated MDT recommended offering adjuvant chemotherapy to patients with a low RS, indicating that these patients are unlikely to benefit from chemotherapy. Overall, based on the use of the RS the decision to offer adjuvant chemotherapy changed in 36% of patients, and ultimately 24 patients (34.7%) would have been spared chemotherapy.

The simulated MDT outcomes were compared to the real life contemporaneous outcomes where the MDT panel had knowledge of Oncotype scores. All patients in the high RS group who were recommended for adjuvant chemotherapy actually received chemotherapy. In the low RS group, 5 out of 24 patients recommended for adjuvant chemotherapy by the simulated MDT underwent chemotherapy in real life.

Using the current NHS list price, the Oncotype DX test costs £2580, which reflects a total cost of £178 020 to test our cohort. From previous studies, the cost of a course of adjuvant chemotherapy in breast cancer is estimated to be between £3866.17 and £4863.70 depending on regimen.^16^, ^17^ In our study, 32 patients were recommended chemotherapy by the MDT, but only nine patients had an RS > 30. Therefore, the potential saving in chemotherapy costs is £88921.91 to £111865.10.

Excluding patients with favourable PREDICT scores from Oncotype testing may improve cost effectiveness. Omitting the 25 patients that had a predicted survival benefit of ≤2% after the addition of adjuvant chemotherapy (and who the simulated MDT did not recommend chemotherapy to) would yield a cost of £113 520 to Oncotype test the remainder of patients, and is more comparable to the chemotherapy savings. However, one patient in the favourable PREDICT score cohort had a RS > 30 and would have potentially missed out on chemotherapy.

## 4 DISCUSSION

Our study shows that the Oncotype DX test changed the decision to offer adjuvant chemotherapy in more than one‐third of patients with ER+ HER2‐ tumours and 1–3 involved nodes. In the vast majority of cases, the MDT recommended adjuvant chemotherapy to patients with low RS scores. This is broadly in keeping with other studies looking at treatment decisions in node‐negative breast cancer.^6^, ^18^, ^19^

Few published studies have examined the role of Oncotype Dx testing in the node positive setting. Interim results of a UK based trial show that the decision to administer adjuvant chemotherapy changed in 74% of patients, sparing them from chemotherapy.^20^ A US‐based study found that RS was an independent predictor of chemotherapy recommendation in 1–3 node positive patients.^21^

Similar to other studies, we found that the majority of node‐positive patients have a low‐risk genomic signature as assessed by the Oncotype Dx test. Despite this, over 50% of patients in the low risk group with an RS <18 were offered adjuvant chemotherapy by the MDT.

A significant proportion of patients fell into the intermediate‐risk group with an RS 18–30, and there are ongoing trials to determine whether there is a subgroup of these patients who may benefit from adjuvant chemotherapy. The patients in this group represent a real challenge for breast MDTs. The highly anticipated results from the RxPONDER trial will help inform the role of adjuvant chemotherapy in patients with RS <25.

There is also variability in the RS cut‐off values used amongst different studies. The initial SWOG‐8814 trial used a definition of RS > 30 to define patients at high risk of recurrence,^10^ however, data suggests that decisions about adjuvant chemotherapy are being made using the inclusion criteria of the RxPONDER trial with a lower threshold of 25.^21^ Further studies will be needed to robustly define high and low‐risk groups based on RS.

We found a moderately positive correlation between estimated additional 10‐year overall survival benefit based on PREDICT and RS. To the best of our knowledge, this is the first to examine this relationship in node‐positive patients. Previous studies in node‐negative patients have shown a correlation between RS and low‐risk patients, but discordance in high‐risk patients.^22^, ^23^

This study is one of the very few showing real‐life data on the effect of Oncotype test on adjuvant chemotherapy decisions in 1–3 node positive patients. The management of micrometastases or isolated tumour cells in the axilla remains contentious^24^, ^25^ and therefore were excluded from our study. In patients with four or more lymph nodes involved, most MDTs would recommend adjuvant chemotherapy without the need to resort to genetic assessment tests such as Oncotype. By excluding these two categories of patients, we avoid confounding factors into our data.

There are some limitations in this study. Firstly, a relatively small number of node positive patients were tested and the study was retrospective in nature. There is also selection bias, as the contemporaneous MDT selected patients for Oncotype DX testing on a case by case basis where the decision to offer adjuvant chemotherapy was uncertain and genomic testing was felt to aid decision making.

Additionally, our study did not measure the oncologic or long‐term outcomes in this group of patients as our main focus is on assessing the impact of Oncotype on the decision to offer adjuvant chemotherapy.

Our present study does not show clear‐cut cost‐effectiveness in node‐positive patients, which had been reported by other studies.^26^ However, we have not included the potential costs of toxicities incurred by patients who would unnecessarily undergo chemotherapy (i.e., low RS patients). Additionally, our data does support targeted testing of node‐positive patients; including omitting testing for patients with favourable PREDICT scores that do not predict much benefit from the addition of adjuvant chemotherapy. Omitting these patients and those assessed as low risk by traditional clinicopathological features^27^may improve cost‐effectiveness.

Another limitation of our study is that patient preferences were not taken into account in the cost effectiveness of oncotype testing and chemotherapy usage.

The strengths of this study are that it reflects the real‐life practice and decision making by using actual patient data with members of the simulated MDT panel blinded to the RS to minimise bias. Our study shows that the use of Oncotype DX in selected node positive patients reduces adjuvant chemotherapy use by objectively identifying those patients with a high risk of recurrence.

The use of gene assays in the management of node‐negative breast cancer has been enshrined in multiple national and international guidelines. NICE guidelines have recommended Oncotype DX testing as an option for node‐negative patients with intermediate‐risk of recurrence to help aid decisions about adjuvant chemotherapy.^28^ As more robust prospective data supporting Oncotype testing in node positive patient emerges it will help inform the future revision of these guidelines.^13^

Advances in modern oncology with the increasing use of targeted therapies and novel prognostic markers will ultimately result in more personalised approaches to the treatment of breast cancer patients. Gene‐based assays such as Oncotype DX provide additional valuable information on individual tumour biology and prognosis. Given the short and long term toxicity associated with adjuvant chemotherapy, it is imperative to identify patients who have a high risk of recurrence and therefore most likely to benefit, as well as identify low‐risk patients in whom chemotherapy can be safely omitted. Our study shows that decision‐making based solely on clinicopathological data may lead to overtreatment of node positive patients. However, prospective randomised trial data is still lacking. The results of ongoing trials, such as RxPonder, are eagerly anticipated to fully inform the role of Oncotype DX testing in node positive patients.

## CONFLICT OF INTEREST

The authors have stated explicitly that there are no conflicts of interest in connection with this article.

## AUTHOR CONTRIBUTIONS

All authors had full access to the data in the study and take responsibility for the integrity of the data and the accuracy of the data analysis. **Mohamed Rabie:** Data curation (supporting); formal analysis (supporting); software (supporting); writing – review and editing (supporting). **Konstantinos Geropantas:** Data curation (equal); investigation (equal); writing – review and editing (equal). **Susanna Alexander:** Data curation (equal); formal analysis (equal); writing – review and editing (equal). **Simon Pain:** Data curation (equal); formal analysis (equal); writing – review and editing (equal). **Mina Youssef:** Conceptualization (equal); data curation (equal); formal analysis (equal); investigation (equal); methodology (equal); project administration (equal); writing – review and editing (equal).

## ETHICAL STATEMENT

Our study was registered as a clinical audit and did not require any formal ethical approval.

## Supporting information


**Figure S1** Table showing breakdown of MDT decisions for adjuvant chemotherapy stratified by PREDICT score survival benefit and RS.

## Data Availability

The data that support the findings of this study are available from the corresponding author upon reasonable request.
