# Supplementary material for: The impact of Oncotype DX testing on adjuvant chemotherapy decision making in 1–3 node positive breast cancer
Source: Cancer Rep (Hoboken). 2021 Oct 19;5(8):e1546. doi: 10.1002/cnr2.1546 (PMC9351646; doi:10.1002/cnr2.1546)
Supplement: Supplementary file 1 — Figure S1 Table showing breakdown of MDT decisions for adjuvant chemotherapy stratified by PREDICT score survival benefit and RS. [file CNR2-5-e1546-s001.docx]

|  | Low RS | | High RS | |
| --- | --- | --- | --- | --- |
|  | MDT decision for chemo | | MDT decision for chemo | |
| PREDICT score benefit | Yes | No | Yes | No |
| <2% | 0 | 14 | 0 | 0 |
| 2-5% | 14 | 22 | 3 | 1 |
| >5% | 10 | 0 | 5 | 0 |

Supplementary Figure 1. Table showing breakdown of MDT decisions for adjuvant chemotherapy stratified by PREDICT score survival benefit and RS.
